# Patterns and correlates of mis-implementation in state chronic disease public health practice in the United States

**DOI:** 10.1186/s12889-020-10101-z

**Published:** 2021-01-28

**Authors:** Margaret M. Padek, Stephanie Mazzucca, Peg Allen, Emily Rodriguez Weno, Edward Tsai, Douglas A. Luke, Ross C. Brownson

**Affiliations:** 1 Prevention Research Center, Brown School at Washington University in St. Louis, 1 Brookings Drive, Campus Box 1196, St. Louis, MO 63130 USA; 2 Department of Surgery, Division of Public Health Sciences, and Alvin J. Siteman Cancer Center, Washington University School of Medicine, Washington University in St. Louis 1 Brookings Dr, St. Louis, MO 63130 USA; 3 Center for Public Health System Science, Brown School at Washington University in St Louis, St Louis, MO USA

**Keywords:** Mis-implementation, Evidence-based public health, Chronic disease, Health departments

## Abstract

**Background:**

Much of the disease burden in the United States is preventable through application of existing knowledge. State-level public health practitioners are in ideal positions to affect programs and policies related to chronic disease, but the extent to which mis-implementation occurring with these programs is largely unknown. Mis-implementation refers to ending effective programs and policies prematurely or continuing ineffective ones.

**Methods:**

A 2018 comprehensive survey assessing the extent of mis-implementation and multi-level influences on mis-implementation was reported by state health departments (SHDs). Questions were developed from previous literature. Surveys were emailed to randomly selected SHD employees across the Unites States. Spearman’s correlation and multinomial logistic regression were used to assess factors in mis-implementation.

**Results:**

Half (50.7%) of respondents were chronic disease program managers or unit directors. Forty nine percent reported that programs their SHD oversees sometimes, often or always continued ineffective programs. Over 50% also reported that their SHD sometimes or often ended effective programs. The data suggest the strongest correlates and predictors of mis-implementation were at the organizational level. For example, the number of organizational layers impeded decision-making was significant for both continuing ineffective programs (OR=4.70; 95% CI=2.20, 10.04) and ending effective programs (OR=3.23; 95% CI=1.61, 7.40).

**Conclusion:**

The data suggest that changing certain agency practices may help in minimizing the occurrence of mis-implementation. Further research should focus on adding context to these issues and helping agencies engage in appropriate decision-making. Greater attention to mis-implementation should lead to greater use of effective interventions and more efficient expenditure of resources, ultimately to improve health outcomes.

**Supplementary Information:**

The online version contains supplementary material available at 10.1186/s12889-020-10101-z.

## Background

Recently, there has been an increasing emphasis in public health practice on use of evidence-based decision-making (EBDM) in chronic disease prevention and control [1, 2]. The need to protect and improve the health and well-being of an entire population, rather than the individual, is the basis of public health programs that are commonly delivered through state level agencies and their local partners in the United States. While the field has made strides in the expected use of evidence-based practices and programs, there is still a gap in the way decision-making practices occur related to ending non-evidence based programs and continuing evidence-based programs (EBPs) [3, 4]. Since much of chronic disease burden is preventable [5–7], gaps in delivery of EBPs hinders effective public health practice to improve health.

The mechanisms behind mis-implementation are an important area of inquiry for public health practitioners and researchers [8–10]. The term mis-implementation refers to the inappropriate termination of evidence-based programs or the inappropriate continuation of non-evidence based programs [8]. An example of inappropriate termination of an evidence-based policy in the United States is notable with the rollback of Bush and Obama era healthy school lunch standards [11], which were relaxed despite evidence they increased school-aged children’s consumption of healthy foods [12]. Alternately, an example of inappropriate continuation of non-evidence-based programs is the continued use of health fairs for community screenings, interventions and education. While they may help increase visibility of services to subsets of the community, there is limited evidence that they increase screening follow-up, enhance sustained health knowledge, or improve health outcomes [13, 14]. Previous work in this area suggests that between 58 and 62% of public health programs are evidence-based [15, 16]. However, only 37% of chronic disease prevention staff in state health departments reported programs are often or always discontinued when they should continue [10]. These studies set a baseline for mis-implementation context but did not further explore the contributing factors to these decision-making processes and did not assess the degree to which mis-implementation is occurring in chronic disease public health practices.

Exploring the evidence-based decision-making (EBDM) and related literature suggests that a mix of individual, organizational, agency and policy related factors are at play in organizational decision-making, including whether or not to begin or continue implementing programs, and program outcomes [1, 10]. EBDM which is an approach to decision-making that combines the appropriate research evidence, practitioner expertise, and the characteristics, needs, and preferences of the community, can have a significant impact on health-related outcomes [1, 17]. Specifically, leadership support in applying EBDM frameworks can enhance an organization’s capacity for improved public health practices [1, 18, 19]. Concurrently, contextual factors, cost burden and characteristics of early adopters are also factors in mis- implementation outcomes [16, 20]. These concepts support the factors that inform our original mis- implementation framework [8]. In a cross-sectional U.S. study of local health departments, higher perceived organizational supports for EBDM were associated with lower perceived frequency of inappropriate continuation [21]. In cross-country comparisons of mis-implementation involving Australia, Brazil, China, and the United States, leadership support and political contexts were common factors in whether chronic disease programs continued or ended inappropriately across four countries [9].

State health departments (SHDs) are a significant driver of public health programs within the United States. Most federal funds for chronic disease prevention are directed through state health departments, and they provide resources and guidance to local level implementation of public health programs [22]. This dynamic of the SHD as the pass-through organization means that their organizational dynamics are key in the successful outcomes of these programs. A common delivery structure in the U.S. is for local public health agencies and community-based organizations to design how they will implement topic-specific programs as they respond to SHD requests for proposals. Another delivery structure is contractual relationships generated by the SHDs in which local agencies choose from a menu of programmatic approaches to chronic disease prevention provided by the SHD. And while an estimated $1.1 billion dollars flow through state public health chronic disease and cancer prevention programs annually, a majority of these funds focus on secondary prevention (e.g., cancer screening), leaving a scarcity for primary prevention resources [23, 24]. With this scarcity in prevention funding, it is essential that every dollar being directed towards chronic disease programs have maximum impact.

This study seeks to: 1) assess the extent to which mis-implementation of chronic disease programs is occurring in state health departments, and 2) identify the most important factors associated with mis-implementation among programs overseen by SHDs [8, 25].

## Methods

This study is a cross-sectional assessment of decision-making practices within state health departments. We surveyed current SHD employees across the U.S. to gather quantitative data to identify the perceived frequency and correlates of mis-implementation within SHD chronic disease units. Human subjects approval was obtained from the Washington University in St. Louis Institutional Review Board (#201812062).

### Survey development

To develop a survey informed by the literature and addressing knowledge and survey gaps, we undertook an extensive literature review. Survey development was also guided by the study team’s previously described conceptual framework to ensure measures included EBDM skills, organizational capacity for EBDM, and external influences such as funding and policy climate [8].

A literature review of several databases (i.e., PubMed, SCOPUS, Web of Science) was conducted to search for existing survey instruments regarding organizational decision-making. Identified measures were summarized according to setting, audience, psychometric properties, and survey question themes. From our review of 63 surveys, we ended up selecting items from 23 measures to examine in relation to our conceptual framework [8–10, 18, 26–40]. Questions pertaining to political influence and mis-implementation decision-making were iteratively developed and refined as there was little published literature available at the time to inform these questions. Drafts for questions in each domain (individual skills, organizational/agency capacity, mis-implementation decision-making, external influences) were updated, and underwent three separate reviews by the study team and a group of practitioner experts to develop a final draft of the study instrument. Since the survey had not been previous validated, the final draft survey underwent cognitive response testing with 11 former SHD chronic disease directors. Reliability test-retest of the revised draft with 39 current SHD chronic disease unit staff found consistency in scores and only minor changes to the survey were needed.

### Measures

Survey measures addressed the following topics: participant demographic characteristics, EBDM skills, perceived frequency of mis-implementation, reasons for mis-implementation, perceived organizational supports for EBDM, and external influences. External influences included perceived governor office and state legislative support for evidence-based interventions (EBIs), and perceived importance of multi-sector partnering. Exact item wording is provided in the national survey located in Additional file 1. Survey questions for EBDM skills, organizational supports, and external influences consisted of 5-point Likert scale responses. Response options ranged from either “Strongly Disagree to Strongly Agree” or “Not at all” to “Very great extent”.

Perceived frequency of mis-implementation was assessed with two questions: “How often do effective programs, overseen by your work unit, end when they should have continued”; and “How often do ineffective programs, overseen by your work unit, continue when they should have ended.” The response options were: never, rarely, sometimes, often, and always. These variables will subsequently be referred to as inappropriate termination and inappropriate continuation, respectively.

### Participants

Participants for the survey were recruited from the National Association of Chronic Disease Directors (NACDD) membership list. The NACDD membership lists consists of SHD employees working in their respective chronic disease units. Participants were randomly selected from the membership roll after individuals from territories and non-qualifying positions (administrative support & financial personnel) were removed. Emails were sent out in June 2018 inviting a randomly selected sample of 1239 members to participate in a Qualtrics online survey. Participants were offered the option of a $20 Amazon gift card or to have us make a donation to a public health charity of their choosing. A follow-up email was sent two weeks after the initial email with a reminder phone call a week later. Non-respondents could have received up to three reminder emails and two reminder voicemails or a single phone conversation to address questions. There was no ability to directly compare non-respondents with respondents given the lack of key characteristics (e.g., role in the agency, years working in the agency) in our initial list for sampling. The online survey closed at the end of August 2018.

### Data cleaning and analysis

Respondents who answered any of the questions beyond demographic questions were included in the sample. State-level variables, such as population size, funding from the Centers for Disease Control and Prevention (CDC) (the major funding source for state chronic disease control), and state governance type, were added to the data set from other publically available datasets such as the CDC grant funding profile, Association of State and Territorial Health Officials (ASTHO) State Profiles^1^ and Public Health Accreditation Board data [23, 25, 41, 42]. Dichotomized versions of Likert scale variables were created given the limited distribution of responses across the original scale and to facilitate interpretation. Responses that included *Agree or Strongly Agree* were coded as 1 while all other remaining responses were coded as 0.

Descriptive statistics were calculated for all variables in SPSS version 26. To assess associations, a Spearman’s correlation was calculated between each non-dichotomized mis-implementation variables and the individual demographic characteristics, individual skills, organizational capacity for EBIs and external factors. Multinomial logistic regression was then used to assess how variables were predictive of mis-implementation outcomes. The dependent variables (inappropriate termination & inappropriate continuation) were re-categorized to 1) often/always 2) sometimes and 3) never/rarely (reference category). Multinomial regression was used as the assumption of proportional odds was violated with an ordinal regression. The independent variables were dichotomized (as described above). Two separate models were fit: the first assessing inappropriate termination among programs overseen by SHDs and the second assessing inappropriate continuation among programs overseen by SHDs. We decided two separate models were appropriate as inappropriate termination and inappropriate continuation are two different phenomena within the overall mis-implementation concept. An initial model for each of the two dependent variables was run for each domain with all their respective variables included. All variables shown to be significant in these first runs of the model were then added to a final version of each model (inappropriate termination and inappropriate continuation).

## Results

### Demographic characteristics

The final response rate was 48.3% (*n*=643). There were respondents from every state, but the number of responses per state was not proportional to state population size. In the interest of confidentiality, responses were grouped by ASTHO defined regions [41], and there was a relatively even distribution of participants across regions (Table 1). Half (50.7%) of the respondents were chronic disease program managers and on average had been in their position for over six years. Most respondents worked across multiple health areas with cancer as the most represented program area. Thirty-five percent of respondents had a master’s or higher degree related to public health.

**Table 1:** Participant characteristics of U.S. SHD employees in chronic disease prevention units, 2018 survey (*N*=643)

State characteristics	
Regions^a^	N (%)
New England	106 (16.5)
South	147 (22.9)
West	104 (16.2)
Mountains/Midwest	149 (23.2)
Mid-Atlantic & Great Lakes	137 (21.3)
**State Size (population)**^a^	
Small (< 2.1 million)	178 (27.7)
Medium (2.1–6.1 million)	224 (34.8)
Large (> 6.1 million)	217 (33.7)
**Practitioner characteristics**	
**Gender**	
Male	131 (19.8)
Female	528 (79.8**)**
Other gender identity	3 (0.5)
**Age**	
Under 20 years	5 (< 1)
20–39 years	213 (33)
40–59 years	352 (54)
60+	90 (13)
**Race & Ethnicity**^b^	
White	527 (79.5)
Black or African American	74 (11.2)
Asian	37 (5.6)
Hispanic	27 (4.1)
American Indian or Alaska Native	14 (2.1)
Hawaiian or Pacific Islander	6 (0.9)
Other	14 (2.1)
**Any Public Health Education**^c^	263 (39.7)
**Primary Work Area**	
Cancer	91 (14.2)
Cardiovascular	45 (7.0)
Diabetes	38 (5.9)
Obesity	80 (12.4)
Tobacco	68 (10.6)
Work in multiple areas	172 (26.7)
Other (e.g. rural health, asthma, school health, etc.)	149 (23.2)
**Position**	
Program manager or coordinator	326 (50.7)
Director overseeing multiple programs	90 (14.0)
Specialists (e.g. epidemiologist, health educator, statistician, etc.)	205 (33.0)
Other (e.g. Administrative roles)	22 (2.0)

^a^As defined by Association of State and Territorial Health Officials [41]

^b^ Could indicate more than one response

^c^ Formal public health degrees include: BSPH, MPH/MSPH, DrPH or PhD in a Public Health field

### Mis-implementation patterns

When asked “How often do effective programs, overseen by your work unit, end when they should have continued,” 50.7% of respondents indicated sometimes, often or always (Table 2). Respondents were asked to choose the top three reasons for effective programs ending (but not in a ranked order). The most common responses were: funding priorities changed/funding ended (87.6%); support from leaders in your agency changed (38.9%); support from policy makers changed (34.2%) and program was not sustainable (30.2%) (Table 2).

**Table 2:** Reported frequency and reasons for mis-implementation from a survey of U.S., 2018

	Perceived frequency of mis-implementation				
	Never	Rarely	Sometimes	Often	Always
**Inappropriate Termination**					
How often do effective programs, overseen by your work unit, end when they should have continued (*n*=613)	7.8%	36.7%	43.2%	7.5%	0.0%
**Inappropriate Continuation**					
How often do ineffective programs, overseen by your work unit, continue when they should have ended (*n*=604)	9.3%	36.1%	40.1%	7.8%	0.6%
**Most common reasons for ending effective programs**^a^		**% endorsing**			
Funding priorities changed/funding ended		87.6%			
Support from leaders in your agency changed		38.9%			
Support from policy makers changed		34.2%			
Program was not sustainable		30.2%			
Program champions left the agency		24.4%			
Lack of staff capacity to write or manage new grants		21.8%			
Lack of inclusion of partnering organizations		6.7%			
Program not made visible to others		5.8%			
Support from general public changed		2.8%			
Other		2.5%			
Staff lacks public health training		1.7%			
**Most common reasons for continuing ineffective programs**^a^		**% endorsing**			
Funder priorities to maintain program		43.4%			
Policy makers’ request or requirements to continue		42.9%			
Agency leadership requests or requirements to continue		37.9%			
Standard is to maintain the status quo		36.5%			
Advocacy group support		19.4%			
Limited evidence available to support ending programs		14.9%			
Program champion support		14.2%			
Disagreement with alternate approaches		12.9%			
Not cost effective to change programs		10.3%			
Evidence-based practices not available for the setting		9.6%			
Staff morale may be affected if program is ended		5.0%			
Other		3.0%			

^a^Respondents could choose up to 3 reasons so percentages will add up to more than 100%. List is arranged by top responses in descending order of frequency a reason was selected; complete questions are shown in Additional file 1

Regarding inappropriate continuation, when asked “How often do ineffective programs, overseen by your work unit, continue when they should have ended,” 48.5% of respondents indicated sometimes, often or always. Respondents were also asked to choose the top three common reasons for ineffective programs continuing (not in ranked order). The most commons responses were: funder priorities to maintain program (43.4%); policy makers’ request or requirements to continue (42.9%); agency leadership requests to continue (37.9%); and standard is to maintain status quo (36.5%) (Table 2).

### Mis-implementation correlates

The number of years a participant had been working in their current position (*r*= − 0.11), years they had been working at their agency (*r*= − 0.09) and years they had been working in public health (*r*= − 0.10) were shown to have small negative significant correlations with inappropriate continuation (Table 3), meaning more years of experience were associated with lower likelihood of inappropriate continuation. None of the individual skills were shown to have a statistically significant association with either inappropriate termination or inappropriate continuation. All of the organizational capacity variables were shown to have a small negative significant association with both mis-implementation variables, meaning higher perceived organizational capacity was associated with lower perceived frequency of mis-implementation (Table 3). External variables related to lawmakers’ priorities and support were shown to have small negative significant, associations with both the inappropriate termination and inappropriate continuation variable.

**Table 3:** Practitioner, Organization, and External Correlates of Mis-Implementation in U.S. SHD chronic disease units, 2018 (N=613)

Variable	Mean (Std Dev)	Spearman’s Correlation (r)	
		Inappropriate Termination	Inappropriate Continuation
**Individual Demographics**			
Years working in current position	6.78 (5.54)	0.04	− 0.11**
Years working at agency	11.73 (11.73)	0.01	−0.09*
Years in public health	15.79 (15.79)	0.07	−0.10*
**Individual Skills**^a^			
I am knowledgeable about EBPH processes	4.39 (0.60)	0.03	−0.00
I have the skills to modify EBIs between one priority population to another	4.30 (0.96)	0.06	−0.01
I have the ability to lead efforts in EBPH in my work unit	3.67 (0.95)	0.01	−0.00
I have the skills to manage program and policy change in my work unit	3.62 (0.95)	0.01	0.01
I have the skills to effectively communicate the value of EBIs to leaders in my agency	3.69 (0.95)	0.01	0.06
I have the skills to effectively communicate information on EBIs to decision makers outsider my agency	3.59 (0.90)	0.15	0.06
**Organizational Capacity for EBDM**			
In my agency, the number of layers of authority impede decisions about program continuation or ending	3.52 (0.92)	0.15**	0.27**
*To what extent do you agree with the statements regarding your agency and work unit*^a^			
My agency uses quality improvement processes	3.72 (1.01)	−0.19**	− 0.20**
My work unit plans for sustainability of programs	3.81 (0.95)	−0.22**	−0.19**
My work unit uses economic evaluation in its decision-making process	3.21 (1.06)	−0.13**	−0.26**
My work unit chooses EBPs because they work in similar populations to those we serve	4.06 (0.84)	−0.11**	−0.22**
My work unit’s leaders are competent at managing change	3.71 (1.06)	−0.21**	−0.30**
There are champions in my work unit who strongly support EBPs	4.24 (0.82)	−0.08*	−0.18**
*Please indicate the extent to which you agree with each statement*^b^			
Leadership in my work unit has developed a plan to implement EBIs	3.59 (1.03)	−0.20**	−0.26**
Leadership in my work unit has removed obstacles to implement EBIs	3.20 (0.99)	−0.24**	−0.26**
Leadership in my work unit recognizes and appreciates employee efforts toward successful implementation of EBIs	3.52 (1.14)	−0.20**	−0.28**
Leadership in my work unit encourages planning for sustainability of programs	3.60 (1.09)	−0.27**	−0.27**
Leadership in my work unit preserves through the ups and downs of implementing EBIs	3.60 (1.94)	−0.24**	−0.25**
Leadership in my work unit supports employee’s efforts to use EBIs	3.89 (1.03)	−0.22**	−0.26**
Leadership in my work unit reacts to critical issues regarding the implementation of EBIs by openly and effectively addressing the problem.	3.31 (0.93)	−0.28**	−0.30**
The extent my agency is willing to make change to enable the use of EBIs	3.37 (0.94)	−0.19**	−0.28**
**External Factors**^a^			
Activities of my work unit fit with priorities of most of our state legislators	3.30 (0.85)	−0.18**	−0.14**
In this past legislative session, most of our state legislators were supportive of EBIs in public health	3.05 (0.81)	−0.19**	−0.22**
Activities of my work unit fit with priorities of the governor’s office	3.48 (0.89)	−0.19**	−0.18**
In the past year, the governor’s office was supportive of EBIs in public health	3.38 (0.89)	−0.14**	−0.19**
It is important for my work unit to develop partnerships with both health and other work sectors to address out state’s health issues.	4.67 (0.54)	0.02	0.02

Boldface indicates statistical significance (*p< 0.05, **p< 0.01)

^a^ 5-point Likert scale used. 1-Strongly Disagree, 5- Strongly Agree ^b^ 5-point Likert Scale used. 1- Not at all, 5- Very great extent

In the final model for inappropriate termination (Table 4) the largest effects were shown for having individual skills to modify EBIs from one priority population to another (OR=3.24; 95% CI=1.19, 8.85); reporting that the number of layers of authority impedes decision-making (OR=3.23; 95% CI=1.61, 7.40); and leadership preserves through ups and downs of implementing EBIs (OR=0.16; 95% CI=0.07, 0.34). In the final model for inappropriate continuation, the largest effects were shown for reporting that the number of layers of authority impedes decision-making (OR=4.70; 95% CI=2.20, 10.04); use of economic evaluation in decision-making (OR=0.35; 95% CI=0.17, 0.73); and leadership competence in managing change (OR=0.26; 95% CI=0.13, 0.53).

**Table 4:** Mis-Implementation Predictors among programs overseen by U.S. state health department chronic disease unit staff, 2018 (N=613)

Independent variable	Dependent Variable Category	OR	95% CI
**Final Inappropriate Termination among programs overseen by SHD Model**			
I have the skills to modify EBIs between one priority population to another	Often/Always Sometimes Never/Rarely	**3.24** 1.25 1 (ref)	1.19, 8.85 0.83, 1.89
Work unit uses the CDC’s Community Guide in its work	Often/Always	1.56	0.66, 3.72
	Sometimes Never/Rarely	**1.73** 1 (ref)	1.10, 2.73
In my agency, the number of layers of authority impede decisions about program continuation or ending	Often/Always	**3.23**	1.61, 7.40
	Sometimes Never/Rarely	1.25 1 (ref)	0.91, 1.83
My agency uses quality improvement processes	Often/Always	0.52	0.27, 1.03
	Sometimes Never/Rarely	**0.49** 1 (ref)	0.34, 0.73
Leadership in work unit perseveres through the ups and downs of implementing EBIs	Often/Always	**0.16**	0.07, 0.34
	Sometimes Never/Rarely	**0.62** 1 (ref)	0.43, 0.89
**Final Inappropriate Continuation among programs overseen by SHD Model**			
In agency, the number of layers of authority impede decisions about program continuation or ending	Often/Always Sometimes Never/Rarely	**4.70** **1.63** 1 (ref)	2.20, 10.04 1.14, 2.34
Work unit uses economic evaluation in its decision-making about programs	Often/Always Sometimes Never/Rarely	**0.35** **0.62** 1 (ref)	0.17, 0.73 0.43, 0.89
Work unit leaders are competent at managing change	Often/Always Sometimes Never/Rarely	**0.26** **0.57** 1 (ref)	0.13, 0.53 0.38, 0.87
The agency is willing to make changes to enable use of EBIs	Often/Always Sometimes Never/Rarely	0.57 **0.54** 1 (ref)	0.27, 1.23 0.37, 0.78

Boldface indicates statistical significance (*p*< 0.05)

The survey items for independent variables were asked on a 5-point scale (strongly disagree to strongly agree) and were dichotomized for model specification into strongly agree/agree (1) and neither disagree or agree/disagree/strongly disagree (0). Model fit statistics for model 1 were X^2^(12)= 83.88, *p*< 0.001 and for model 2 were X^2^(8)= 103.11, *p*< 0.001. OR: odds ratio, CI: confidence interval

## Discussion

A set of organization/agency capacity factors demonstrated more consistent association with mis-implementation outcomes than individual skills of staff. These factors demonstrated an inverse relationship with mis-implementation outcomes (e.g., as agency capacity increased, the association with mis-implementation rates decreased). These findings are consistent with our earlier study among US local health departments, which found organizational supports for EBDM were associated with lower perceived frequency of inappropriate continuation [21]. This suggests agency culture and capacity are significant protective factors against mis-implementation in multiple public health organizations rather than the skills of individual staff. Importantly, the agency-level variable reporting that the number of layers of authority impedes decision-making about programs continuation or ending was found to be strongly associated with both inappropriate termination and continuation. This suggests that highly vertical organizations may be more vulnerable to ineffective decision-making around program continuation or ending. Given that state health departments vary widely in their organizational structures, further work is needed to understand how a large number of layers may affect decision making that leads to more frequent use of evidence-based decision making in public health practice [17].

Outside of funding, the primary correlates for inappropriate termination or continuation were changing support from leaders and policymakers. We saw more variability in the reasons for inappropriate continuation versus termination. Inappropriate termination was heavily skewed towards funding priorities changing or ending, which is to be expected given the predominance of state public health programs based on time-limited grant funding [43]. The top four reasons for inappropriate continuation were more spread out across multiple domains. This variability in reasons could demonstrate that the processes that result in an ineffective program continuing may tend to involved multiple domains, but this also allows for more opportunity for modifiability.

The two factors most strongly negatively correlated with inappropriate *continuation* related to leaders’ flexibility were: work unit’s leaders are competent at managing change (*r*=− 0.30); and leadership reacts to critical issues regarding the implementation of EBIs (*r*=− 0.30). The two factors most strongly correlated with inappropriate *termination were*: work unit leadership react to critical issues regarding the implementation of EBIs; work unit leadership encourages planning for sustainability of programs (*r*= − 0.28, − 0.27 respectively). Again, this suggests agency culture and leadership are strong drivers of mis-implementation outcomes but more specifically how leadership can be related to the importance of EBI use and flexibility in program implementation and adaption.

Our findings are largely consistent with the literature in EBDM and have several implications for public health practice. Reviews found organizational climate, leadership support, staff commitment and skills, adequate staffing and low staff turnover, organizational resources, and partnerships affect EBI sustainability [4, 44, 45]. Policy, in the form of legislation and regulation, are also associated with sustainment of programs in community, clinical and social service settings [45]. Engaging community leaders and other policy makers throughout programmatic decision-making can increase likelihood of program sustainment [44]. While de-implementation of ineffective clinical tests or services has been studied, there is sparse parallel literature on de-implementation of ineffective public health programs [8, 46–48]. As our study illustrates, effective public health practice is not solely based on the effectiveness of the programs themselves but also the capacity of the organizations deliver them. Capacity is multi-faceted, and understanding an organization’s culture and hierarchy could reveal more about successful public health program implementation.

### Limitations

Our response rates across states was varied enough that we were not able to study state-level correlates in detail. In the absence of other organizational and administrative data, this study relied on self-report surveys of individual and perceived organizational characteristics. While we asked respondents their level of involvement in decision-making, they were not always in the position to be privy to the reasons about decision-making or they joined the agency after a decision about a program had concluded.

Compared with previous pilot work, perceived frequency of mis-implementation in SHD was higher in this study (36.5% vs 50.7% for inappropriate termination and 24.7% vs 48.5% for inappropriate continuation), although some of this difference may be attributable in part to updates to the mis-implementation survey item definitions and changes in the approach to categorization of responses [9, 10, 21]. In earlier studies, the recoded dichotomized mis-implementation variables only included the often/always response. After examining the distribution of the mis-implementation variables responses, we thought it was important to include the “sometimes” response in categorizing mis-implementation because “sometimes” still captured the phenomena occurring and that excluding it could potentially leave out nuances in the data.

### Future directions

These results provide a first look at factors that may be related to the phenomena of mis-implementation in public health practice. Later phases of this study include eight case studies highlighting lessons learned around mis-implementation and agent-based modeling to identify the dynamic interactions between the individual, organizations and contextual factors and disseminate them back to the state health departments [8]. The results of these qualitative case studies will be available in future publications. These models should provide decision-making tools to better facilitate evidence-based decision making.

There is also a need to explore mis-implementation in other public health settings. While our study focuses on SHDs, local health departments and non-profit settings are significant implementers of public health programs. There is also sparse information on how mis-implementation may vary across public health program areas (e.g., chronic disease, infectious disease, maternal and child health). Additional comparisons of organizational structures across state health departments could also explore the context underlying the “flattening” variable we found as an important correlate of mis-implementation.

## Conclusion

While our understanding of mis-implementation in public health practice is in an early stage, our findings provide practitioners and applied researchers some actionable findings. For example, based on our study and related literature [18, 49, 50]. it appears that efficiency and effectiveness may be gained via flattening of public health agencies along with an organizational culture that supports EBDM. Given the emergence of evidence that chronic diseases are a significant moderating factor in outcome of timely disease concerns i.e. COVID-19 and cancer risk [51, 52], suggestions like these could help maximize dollars spent on public health programs ensuring that appropriate evidence-based programs are contributing to improved health outcomes and benefiting the communities they serve.

## Supplementary Information

**Additional file 1.** Mis-Implementation National Survey.

## Data Availability

The datasets used and analyzed during the current study are available from the corresponding author on reasonable request.

## Abbreviations

SHD: State Health Department
EBDM: Evidence Based Decision-making
EBIs: Evidence-Based Interventions
ASTHO: Association of State and Territorial Health Organizations
EBPs: Evidence based programs.
NACDD: National Association of Chronic Disease Directors

## Footnotes

1 A report on state and territorial public health agencies outlining their structures, activity, financial and workforce resources. The report is updated every 2–3 years [41].
