# Supplementary material for: Patterns and correlates of mis-implementation in state chronic disease public health practice in the United States
Source: BMC Public Health. 2021 Jan 28;21:101. doi: 10.1186/s12889-020-10101-z (PMC7840223; doi:10.1186/s12889-020-10101-z)
Supplement: Supplementary file 1 — Additional file 1. Mis-Implementation National Survey. [file 12889_2020_10101_MOESM1_ESM.docx]

Introduction

Please answer the following questions about your background and that of your agency.

1. How do you best describe your agency/ organization? (Please check only one.)

- State Health Department
- Local Health Department
- Tribal Health Agency
- Coalition
- Advocacy Group
- University or School
- Community-Based Organization
- Voluntary Health Organization (e.g. American Cancer Society)
- Heath Care Facility (e.g. hospital, clinic, Federally Qualified Health Center)
- Other ________________________________________________

1. Which of the following best describes your position? (Please check only one.)

- Program Manager or Coordinator
- Health Educator
- Legislative Liaison
- Behavioral Scientist
- Epidemiologist
- Statistician
- Program Evaluator
- Cancer Registrar
- Community/Public Health Nurse
- Social Worker
- Dietitian or Nutritionist
- Director overseeing multiple programs in a section, bureau or division
- Other ________________________________________________

Please choose from the drop down list to indicate how long you have been in each of the following.

Please round to the nearest year

3) Years working in your current position.

4) Years working in your agency/ organization (any position).

5) Years working in public health practice in total (in any agency, in any position).

6) In which of the following program areas do you primarily work? (Please select only one.)

- Arthritis
- Asthma
- Cancer Prevention and Control
- Cardiovascular Disease
- Diabetes
- Environmental Health
- Healthy Aging
- Minority Health
- Adolescent/Teen Health
- Immunizations
- Rural Health
- Health Equity/ Social Determinants
- Injury and Violence Prevention
- Maternal and Child Health
- Obesity Prevention, Physical Activity, Diet/Nutrition
- Oral Health
- School Health
- Tobacco
- I work primarily across multiple program areas
- Other ________________________________________________

7) Please select your role or focus in the program in which you work. (Check all that apply.)

- Epidemiology and Surveillance (Domain 1)
- Environmental Approaches (Domain 2)
- Health Care System Interventions (Domain 3)
- Community Programs linked to Clinical Services (Domain 4)
- Health Communication
- Health Education and Promotion
- Health Equity/Social Determinants
- Quality Improvement
- Other ________________________________________________

8) What is your gender?

- Male
- Female
- Other gender identity

9) What is your age?

- Under 20 years
- 20-29 years
- 30-39 years
- 40-49 years
- 50-59 years
- 60-69 years
- 70+ years

10) Which degrees/credentials do you hold? (Check all that apply.)

- High School diploma or equivalent
- Associates degree
- Bachelor's degree in any field
- Bachelor's of Science in Nursing
- Certificate in Public Health
- Certificate in Informatics
- Certificate in Tumor Registry
- CHES or MCHES
- MPH or MSPH
- Master's in Informatics/BioInformatics/Health Informatics
- Master's degree in any field
- ARNP
- RN
- MSN
- PNP
- PhD in Nursing
- RD
- Doctoral degree in any field
- PhD, DrPH, ScD in Public Health
- MD, DO, or DDS
- RS (Registered Sanitarian)
- Other ________________________________________________

11) What is your race? (Check all that apply)

- White
- Black or African American
- American Indian or Alaska Native
- Asian
- Native Hawaiian or Pacific Islander
- Other ________________________________________________
- Prefer not to answer

12) Are you of Hispanic origin, such as Mexican American, Latin American, Puerto Rican, Cuban?

- Yes (please specify group) ________________________________________________
- No
- Prefer not to answer

Individual Skills

The next set of questions asks about your individual public health skills as it pertains to the work in your state health department. Please answer these to the best of your ability. If you prefer not to answer a question, you may skip the question.

We define "work unit" as the group of programs in the same administrative unit that include your agency's programs in chronic disease, health promotion, tobacco and other substances, adolescent health, injury and violence prevention, and related areas.

13) Evidence-based public health is defined as the "process of integrating science-based interventions with community preferences to improve the health of populations."
 
I am knowledgeable about evidence-based public health processes.

- Strongly disagree
- Disagree
- Neither agree nor disagree
- Agree
- Strongly agree

14) I have the skills I need to modify evidence-based interventions from one priority population to another.

- Strongly disagree
- Disagree
- Neither agree nor disagree
- Agree
- Strongly agree

15) I have the ability to lead efforts in evidence-based public health in my work unit.

- Strongly disagree
- Disagree
- Neither agree nor disagree
- Agree
- Strongly agree

16) I have the skills to manage program and policy change in my work unit.

- Strongly disagree
- Disagree
- Neither agree nor disagree
- Agree
- Strongly agree

17) I have the skills to effectively communicate the value of evidence-based interventions to leaders in my agency.

- Strongly disagree
- Disagree
- Neither agree nor disagree
- Agree
- Strongly agree

18) I have the skills to effectively communicate information on evidence-based interventions to decision makers outside my agency (such as community leaders, policy makers, elected officials, business leaders).

- Strongly disagree
- Disagree
- Neither agree nor disagree
- Agree
- Strongly agree

19) What is the primary way by which you learn about the latest information on effective interventions? (Please choose one.)

- Government sources (e.g. agency staff, government reports, agency listservs, policy briefs)
- Peer-reviewed sources (e.g. academic journals, conferences, evidence-based repositories)
- Social media (e.g. Facebook, Twitter, LinkedIn)
- Open internet (e.g. search engines)
- Other ________________________________________________

Decision Making on Programs Ending

This section asks for **your** perspectives on decision-making about programs that **ended**.

A **program** is defined here as an organized public health action, such as direct service interventions, community mobilization efforts, policy, system and environmental changes, outbreak and cluster investigations, health communication campaigns, or health promotion programs.

20) From the list, choose up to the 3 roles of people in your organization or state who are the **most involved** in the decision to **end** programs.

- Program Managers
- Bureau/Section/Division Leaders
- Agency-wide Leaders/ Commissioners
- Legislators
- Governor/Governor's Senior Staff
- Other (please specify) ________________________________________________

21) How often are **you** involved in decisions about programs **ending**?

- Never
- Rarely
- Sometimes
- Often
- Always

22) How influential are **your** opinions regarding decisions about programs **ending**?

- Not at all influential
- Slightly influential
- Somewhat influential
- Very influential
- Extremely influential

23) How often do **effective** programs, overseen by your work unit, **end** when they should have **continued**?

- Never
- Rarely
- Sometimes
- Often
- Always

24) How often do **effective** programs, overseen* by local health departments in your state, **end** when they should have **continued**?
 
*Every state has a different structure in which they interact with local health departments. Please answer this question to the best of your knowledge.

- Never
- Rarely
- Sometimes
- Often
- Always

25) How often do **effective** programs, overseen by external organizations (contracted by your State Health Department), **end** when they should have **continued**?

- Never
- Rarely
- Sometimes
- Often
- Always

26) When you think about effective programs ending, what are the **most common** reasons? 

Please choose and rank up to 3 top reasons for programs ending.

| ______ Funding priorities changed/funding ended |
| --- |
| ______ Support from policy makers changed |
| ______ Support from general public changed |
| ______ Support from leaders in your agency changed |
| ______ Program champions left the agency |
| ______ Lack of staff capacity to write or manage new grants |
| ______ Program not made visible to others |
| ______ Program was not sustainable |
| ______ Staff lacks public health training |
| ______ Lack of inclusion of partnering organizations |
| ______ Other (please specify) |

27) Do you know of an example of an **effective** program in your state that **ended**?

- Yes
- No

28) Please describe the **effective** program that ended and why it ended.
 
For example: "A school district's enhanced physical activity program ended after funding was lost."
 
(We remind our participants that all responses are kept confidential and analyzed in the aggregate. Individual responses WILL NOT be shared with employers or others.)

________________________________________________________________

________________________________________________________________

________________________________________________________________

________________________________________________________________

________________________________________________________________

Decision-Making about Programs Continuing

This section asks for your perspectives on decision-making that occurs regarding a program's **continuation**.
 
PLEASE NOTE that many of the questions will look similar to ones on the previous page. However, these questions are addressing the specifics of decision-making to **continue** a program. 

29) From the list, choose up to the 3 roles of people in your organization or state who are the most involved in the decision to continue programs.

- Program Managers
- Bureau/ Section/Division Leaders
- Agency-Wide Leaders/ Commissioners
- Legislators
- Governor/Governor's Senior Staff
- Other (please specify) ________________________________________________

30) How often are **you** involved in decisions to **continue** programs?

- Never
- Rarely
- Sometimes
- Often
- Always

31) How influential are your opinions regarding decisions about programs continuing?

- Not at all influential
- Slightly influential
- Somewhat influential
- Very influential
- Extremely influential

32) How often do **ineffective** programs, overseen by your work unit, **continue** when they should have **ended**?

- Never
- Rarely
- Sometimes
- Often
- Always

33) How often do **ineffective** programs, overseen* by local health departments in your state, **continue** when they should have **ended**? 

*Every state has a different structure in which they interact with local health departments. Please answer this question to the best of your knowledge.

- Never
- Rarely
- Sometimes
- Often
- Always

34) How often do **ineffective** programs, overseen by external organizations (contracted by your State Health Department), **continue** when they should have **ended**?

- Never
- Rarely
- Sometimes
- Often
- Always

35) When you think about **ineffective** programs **continuing**, what are the **most common** reasons? 
 
Please choose and rank up to 3 top reasons for programs continuing.

|  |
| --- |
| ______ Policy makers' requests or requirements to continue |
| ______ Agency leadership requests or requirements to continue |
| ______ Advocacy group support |
| ______ Program champion support |
| ______ Standard is to maintain the status quo/ change is hard |
| ______ Staff morale may be affected if program is ended |
| ______ Disagreement with alternate approaches |
| ______ Limited evidence available to support ending programs |
| ______ Evidence-based practices not available for the setting or population group |
| ______ Funder priorities to maintain program |
| ______ Not cost effective to change programs |
| ______ Other |

36) Do you know of an example of an **ineffective** program in your state that **continued** that should have **ended**?

- Yes
- No

37) Please describe the **ineffective** program that should have ended, and why it was continued.
 
For example: "A substance abuse prevention program aimed at school age children that showed no decrease in use of alcohol or other drugs continued because it gave the schools a positive public image and did not cost the schools money." 
 
(We remind our participants that all responses are kept confidential and analyzed in the aggregate. Individual responses WILL NOT be shared with employers or others.)

________________________________________________________________

________________________________________________________________

________________________________________________________________

________________________________________________________________

________________________________________________________________

Organization/ Agency Capacity

The following questions ask about your organization. For these questions we want you to think about your work unit.

38) Does your work unit use the CDC Community Guide in its work?

- No
- Yes, sometimes
- Yes, often
- I am not familiar with the Community Guide

39) In my agency, the number of layers of authority impede decisions about program continuation or ending.

- Strongly disagree
- Disagree
- Neither agree nor disagree
- Agree
- Strongly agree

40) To what extent do you agree with the statements below regarding your agency and work unit?

|  | Strongly disagree | Disagree | Neither agree nor disagree | Agree | Strongly agree |
| --- | --- | --- | --- | --- | --- |
| My agency uses quality improvement processes such as LEAN, Plan-Do-Study-Act, etc. |  |  |  |  |  |
| My work unit plans for sustainability of programs. |  |  |  |  |  |
| My work unit includes economic evaluation in its decision-making about programs. |  |  |  |  |  |
| My work unit chooses evidence-based programs because they work in populations similar to those we serve. |  |  |  |  |  |
| My work unit's leaders are competent at managing change. |  |  |  |  |  |
| There are champions in my work unit who strongly support evidence-based programs. |  |  |  |  |  |

41) The following questions asks about leadership in your work unit. Please indicate the extent to which you agree with each statement.

Leadership in my work unit....

|  | Not at all | Slight extent | Moderate extent | Great extent | Very great extent |
| --- | --- | --- | --- | --- | --- |
| Has developed a plan to implement evidence-based interventions. |  |  |  |  |  |
| Has removed obstacles to the implementation of evidence-based interventions. |  |  |  |  |  |
| Recognizes and appreciates employee efforts toward successful implementation of evidence-based interventions. |  |  |  |  |  |
| Encourages planning for sustainability of programs. |  |  |  |  |  |
| Perseveres through the ups and downs of implementing evidence-based interventions. |  |  |  |  |  |
| Supports employees' efforts to use evidence-based interventions. |  |  |  |  |  |
| Reacts to critical issues regarding the implementation of evidence-based interventions by openly and effectively addressing the problem. |  |  |  |  |  |

42) To what extent is your agency willing to make changes (e.g. enhance workforce training, seek out new partners) to enable the use of evidence-based interventions?

- Not at all
- Slight extent
- Moderate extent
- Great extent
- Very great extent

External Influences

You're almost finished!

In this final section, we will ask about external factors and relationships that may affect decision-making regarding programs.

43) The activities of my work unit fit with the priorities of most of our state legislators.

- Strongly disagree
- Disagree
- Neither agree nor disagree
- Agree
- Strongly agree

44) In this past legislative session, most of our state legislators were supportive of evidence-based interventions in public health.

- Strongly disagree
- Disagree
- Neither agree nor disagree
- Agree
- Somewhat agree

45) The activities of my work unit fit with the priorities of the governor’s office.

- Strongly disagree
- Disagree
- Neither agree nor disagree
- Agree
- Strongly agree

46) In the past year, the governor's office was supportive of evidence-based interventions in public health.

- Strongly disagree
- Disagree
- Neither agree nor disagree
- Agree
- Strongly agree

47) It is important for my work unit to develop partnerships with both health and other work sectors to address our state's health issues.

- Strongly disagree
- Disagree
- Neither agree nor disagree
- Agree
- Strongly agree

48) Which types of health organizations does your work unit currently collaborate with? (Check all that apply.)

- Local Health Departments
- Other State Health Departments
- Clinics or Federally Qualified Health Centers (FQHCs)
- Hospitals
- University Schools or Departments focused on health
- Indian Health Service
- Tribal Health Organizations
- Medicaid unit of state agency
- State Medical Associations
- Health Non-Profits (e.g. American Cancer Society, American Heart Association)
- Health Insurance Providers
- Mental Health Services
- Foundations/Public Health Institutes
- Other ________________________________________________

49) For the health organizations your work unit collaborates with, on the next page, please indicate the type of collaboration: 
  (Choose all that apply.)

|  | Exchange information | Work together on activities or projects | My agency provides financial resources | My agency serves a leadership role in the collaboration | My agency is a recipient of financial resources from this organization |
| --- | --- | --- | --- | --- | --- |
| Local Health Departments |  |  |  |  |  |
| Other State Health Departments |  |  |  |  |  |
| Clinics or Federally Qualified Health Centers (FQHCs) |  |  |  |  |  |
| Hospitals |  |  |  |  |  |
| University Schools or Departments focused on health |  |  |  |  |  |
| Indian Health Service |  |  |  |  |  |
| Tribal Health Organizations |  |  |  |  |  |
| Medicaid unit of state agency |  |  |  |  |  |
| State Medical Associations |  |  |  |  |  |
| Health Non-Profits (e.g. American Cancer Society, American Heart Association) |  |  |  |  |  |
| Health Insurance Providers |  |  |  |  |  |
| Mental Health Services |  |  |  |  |  |
| Foundations/Public Health Institutes |  |  |  |  |  |
| Other |  |  |  |  |  |

50) Which sectors or organizations **outside** of the health sector does your work unit currently collaborate with? (Check all that apply.)

- University Schools or Departments that are non-health focused (e.g. Engineering, Arts)
- Pre K-12 Education/ Youth Programs (e.g. Schools, Youth Development Agencies, YMCA)
- Media, Communications, Public Relations Organizations
- Community Development Organizations
- Social Services other than Medicaid
- Businesses
- Parks and Recreation Departments
- Housing
- City Planning or Transportation Agencies
- Justice System
- State Commissions, Special Councils
- Tribal Agencies
- Other State Agencies
- Other ________________________________________________

51) For the sectors or organizations your work unit collaborates with, on the next page, please indicate the type of collaboration: 
  (Choose all that apply.)

|  | Exchange Information | Work together on activities or projects | My agency provides financial resources | My agency serves a leadership role in the collaboration | My agency is a recipient of financial resources from this organization |
| --- | --- | --- | --- | --- | --- |
| University Schools or Departments that are non-health focused (e.g. Engineering, Arts) |  |  |  |  |  |
| Pre K-12 Education/ Youth Programs (e.g. Schools, Youth Development Agencies, YMCA) |  |  |  |  |  |
| Media, Communications, Public Relations Organizations |  |  |  |  |  |
| Community Development Organizations |  |  |  |  |  |
| Social Services other than Medicaid |  |  |  |  |  |
| Businesses |  |  |  |  |  |
| Parks and Recreation Departments |  |  |  |  |  |
| Housing |  |  |  |  |  |
| City Planning or Transportation Agencies |  |  |  |  |  |
| Justice System |  |  |  |  |  |
| State Commissions, Special Councils |  |  |  |  |  |
| Tribal Agencies |  |  |  |  |  |
| Other State Agencies |  |  |  |  |  |
| Other |  |  |  |  |  |
